# Coalitions and Public Action in the Reshaping of Corporate Responsibility: The Case of the Retail Banking Industry

**DOI:** 10.1007/s10551-020-04529-x

**Published:** 2020-05-25

**Authors:** Marta de la Cuesta-González, Julie Froud, Daniel Tischer

**Affiliations:** 1 Departamento de Economía Aplicada, Facultad de Económicas y Empresariales de la UNED, C/ Senda del Rey 11, 28040 Madrid, Spain; 2 Alliance Manchester Business School, University of Manchester, Booth St West, Manchester, M156PB UK; 3 School of Management, 2.06 Howard House, Queen’s Avenue, Bristol, BS8 1SD UK

**Keywords:** Corporate responsibility, Industry corporate responsibility, Business ethics, Retail banking, Stakeholder negotiation, Bank business model, Financial services, Spain, UK

## Abstract

This paper addresses the question of whether and how public action via civil society and/or government can meaningfully shape industry-wide corporate responsibility (ICR) behaviour. We explore how, in principle, ICR can come about and what conditions might be effective in promoting more ethical behaviour. We propose a framework to understand attempts to develop more responsible behaviour at an industry level through processes of negotiation and coalition building. We suggest that any attempt to meaningfully influence ICR would require stakeholders to possess both power and legitimacy; moreover, magnitude and urgency of the issue at stake may affect the ability to influence ICR. The framework is applied to the retail banking industry, focusing on post-crisis experiences in two countries—Spain and the UK—where there has been considerable pressure on the retail banking industry by civil society and/or government to change behaviours, especially to abandon unethical practices. We illustrate in this paper how corporate responsibility at the sector level in retail banking is the product of context-specific processes of negotiation between civil society and public authorities, on behalf of customers and other stakeholders, drawing on legal and other institutions to influence industry behaviour.

## Introduction

The scrutiny by governments, non-government organisations and civil society that followed the Great Financial Crisis highlighted how banks can abuse their position as powerful economic intermediaries in pursuit of shareholder value creation. In particular, banks were observed to engage in value extraction through fees or speculative activities disembedded from the real economy (Muellerleile 2013) and have collectively resisted calls for change, despite significant and sustained national and international uproar about unethical practices. This is important for two reasons in relation to the wider importance of banking in everyday economic and social life. First, as providers of banking, credit, insurance and other services for small- and medium-sized business, retail banks fulfil important intermediary and enabling functions (Froud et al. 2017). Second, access to finance through bank accounts and other financial services is essential for citizens’ economic and social participation (Schmidt et al. 1999; Allen and Santomero 1998). In this sense, retail banking is a foundational infrastructure (Froud et al. 2018). Yet instead of ensuring this basic functionality, banks’ actions to defend profitability have had deleterious effects on their customers, including mis-selling of financial products, collapse of small business lending, mortgage foreclosure and retail branch closures (Froud et al. 2017; Paulet et al. 2015; Vives-Miró and Gutiérrez 2017). These behaviours are contrary to those we might expect from banks as socially responsible actors; and they are in contrast to the positive impact banks can have on financial inclusion, economic and social development (Fernández-Olit and de la Cuesta-González 2014).

In principle, curbs on unethical bank behaviour could result from government action, investor-led initiatives or other pressures from external actors, whereby organisations are compelled or at least encouraged to meet wider stakeholder interests. Governments have tended to see this as the role of investors, and many businesses (including banks) have adopted investor-led corporate social responsibility (CSR) initiatives to address expectations related to broader impacts on society, the economy and the environment (de Bakker et al. 2013; Doh and Guay 2006; Schaltegger and Burritt 2010; Steurer 2010). This positivist or instrumental approach to CSR recognises stakeholder demands for the primary purpose of sustaining or improving profitability and shareholder value (Esteban-Sánchez et al. 2017) but leaves limited opportunity to meaningfully alter business models or the ethical foundations of business (Sternberg 2011). This approach is also centred on easily quantifiable aspects of responsibility (Maniora 2017) more than the impact of their intermediary activity on customer welfare and society (Fernández-Olit and de la Cuesta-González 2014). In this sense, corporate responsibility has often been a documentary artefact accounting for the positive impact of the business on society in annual accounts or standalone reports rather than providing meaningful accounts of ethical business behaviour (Coupland 2006, p. 3).

Such reporting therefore becomes a way of recognising aspects of behaviour that a firm can address—employee diversity or carbon emission reduction programmes, or additions such as sponsoring activities (Gao and Bansall 2013)—without business ethics effectively incorporated into decisions about the underlying business model (Maniora 2017; Sternberg 2011). It is not surprising then that critical observers suggest these instrumental approaches to CSR represent corporate ‘window dressing’, in which social responsibility is treated as an add-on to core operations (Frankental 2001; Lewis 2016; Schaltegger and Burritt 2010). Such initiatives do not in themselves embed and normalise responsible behaviour. Indeed, the recurrence of socially and economically irresponsible or unethical behaviour by firms individually and collectively raises questions about whether organisation-level actions can provide a meaningful approach to tackle (fundamental) problems characterising an industry (Fernández-Olit et al. 2019).

Drawing on these important themes, this paper considers responsibility initiatives affecting firms across an industry which are the outcome of actions by industry stakeholder coalitions rather than arising through voluntaristic action. Such actions are considerably rarer than instrumental, firm-level CSR, despite efforts by civil society to scale up collaborations to target a whole industry (Den Hond et al. 2014). The benefit of assuming an industry perspective lies in what Beschorner and Hajduk (2017) call ‘the downscaling effect’: within an industry context, responsibility can be substantiated and thus made clear and manageable for companies and their stakeholders. The industry-level corporate responsibility (ICR) framework draws attention to ‘interactions between businesses, NGOs, political actors and other organisations’, (Powell and DiMaggio 1991; Beschorner 2004) which may be more evident at an industry rather than an individual firm level. In the specific case of retail banking considered in this paper, ‘reform’ ambitions emerged before, during and after the crisis, reflecting a significant gap between bank behaviour and expectations of fair and effective financial services held by civil society, private and business customers. Mis-selling of financial products, closure of bank branches (especially in rural areas), failure to lend to small- and medium-sized enterprises (SMEs), as well as irresponsible lending on property have variously emerged as endemic problems requiring sector-level action (Fernández-Olit et al. 2019; Froud et al. 2017).

The paper addresses the question of whether and how public action (via civil society and/or government) can meaningfully affect industry-wide corporate responsibility behaviour. Where problems extend over a sector and challenge the basic function of retail banks from a stakeholder perspective, have actions to improve corporate responsibility been coordinated rather than left to the discretion of individual banks? To explore these issues, we focus on post-crisis experiences in two countries, Spain and the UK. These are chosen because, in both cases, there has been considerable pressure from civil society on the retail banking industry to abandon certain activities (mis-selling, repossessions) or counteract others (failure to lend to SMEs) to curb negative impacts on customers. Here, retail banks are economic and political actors (Matten et al. 2003; Scherer et al. 2006) and corporate responsibilities are primarily viewed as reaction to wider changes in societal institutions (Dubbink 2004) and to civil society ambition to shift corporate attention and resources towards societal challenges (Scherer and Palazzo 2011). Socially responsible bank behaviour is therefore not simply an outcome of immediate stakeholder pressures, but a response to democratic forces to curb corporate power towards enacting public needs (Driver and Thompson 2002; Parker 2002).

Our analysis engages critically with Beschorner et al.’s (2013, p. 26f) conception of ICR as embedded in sector-specific collaborative efforts between firms, government and other stakeholders. Coalition building and alignment of interests are key success factors. However, the emphasis on ICR as collaboration can obfuscate the role of stakeholders in bringing about that change in the absence of industry engagement. Therefore we introduce an alternative view of ICR as a politically charged negotiation between industry and its stakeholders. We choose retail banking to explore these themes because this industry has been overwhelmingly inactive in responding to problems caused collectively by its members. Rather, as explored in this paper, it has been civil society—with public authorities providing necessary though limited support—which has played a key role in recognising problems and engaging retail banks through a process of negotiated responsibility. From a bank perspective, such responsibility is reactive, or even coerced, not developed internally (see, for example, Scholte 2013).

This analysis acknowledges that industries (and firms as their constituent actors) are primarily concerned with their ability to continue with business-as-usual and may not initiate change, especially when profits will be affected. With this in mind, we show that ICR is driven by powerful and legitimate interventions from civil society and government. We focus on these actors not because they are the only possible agents of change, but because they have the capacity to campaign for, or to pressure industry members to adopt more responsible behaviours.

The next part of the paper explores the concept of ICR and the role of interest alignment and coalition building in achieving a change in ethical behaviours. The Spanish and UK retail banking sectors are introduced in the third section, which outlines the extent of corporate responsibility initiatives since 2008. The fourth section then takes two examples to explore the role of civil society in negotiations that eventually led to actions. While notable, the impact of these actions is limited: successful actions have been fragmentary and industry behaviour remains largely reactive, constituting individual redress rather than a more generalised ethical orientation. The implications are considered in the final section.

## Coalitions and Industry-Level Corporate Responsibility

In this section, we outline and develop the concept of industry-level corporate responsibility to explore how stakeholders may be able to negotiate through coalitions and alignments of interests to affect the operation of firms in an industry. The analysis draws on the literature to address two questions: first, how in principle ICR might come about, either through industry leadership or coalition building by stakeholders, before exploring resistances to change; and, second, what conditions might allow some actions to be more effective, including the importance of power, legitimacy, magnitude and urgency.

### Industry Corporate Responsibility: From Proactive to Reactive Agendas

An industry-level approach to responsibility represents a relatively new understanding of how firms collectively engage with stakeholder interests on a voluntary basis or under compulsion. Beschorner et al.’s (2013, p. 26f.) ICR framework draws attention to ‘interactions between businesses, NGOs, political actors and other organisations’, to secure the adoption of corporate responsibility policies as a specific ‘organisational field’ (Powell and DiMaggio 1991; Beschorner 2004). For this to be successful, business, civil society and government actors necessarily share assumptions about what corporate responsibility means in a specific industry context. Beschorner et al. (2013) argue that, prior to the adoption of ICR, key stakeholders (customers, worker organisations, civil society) collaborate and negotiate the type and extent of the policies, though they retain an industry-centric view, with stakeholders taking second stage. More recently, Beschorner and Hajduk (2017) advocate a cultural perspective to business ethics—*cultural business ethics*—within which CR initiatives are linked to industry-specific cultural contexts and institutional logics. Here they invoke the notion of the organisational field to situate concrete action through network interactions between stakeholders and core businesses to reduce bias towards the company.

It is helpful to view ICR as an interactive political process that requires negotiation, rather than one that is either top down or bottom up (Dunning 2004), or indeed a combination of both (Behrman 2004). However, the framing by Beschorner et al. (2013) and Beschorner and Hajduk (2017) creates an overly harmonious impression of this process, where, following debates and negotiation, participants can agree on appropriate industry actions. This in turn assumes that agendas are meaningfully represented by stakeholder and industry-led coalitions. A firm’s ability to act is constrained by both the shared interests enshrined in trade association membership and the associations’ own interests, which can have both positive and negative social outcomes (Marques 2017).^1^ Moreover, industry-led coalitions are likely to have diverse motivations, as indicated by Grayson and Jane (2013): they may proactively engage with social inequalities to pre-empt negative impact on industry and limit government oversight; or they may be a prerequisite for market access. Such coalitions can also be more instrumental in creating visibility for firms and industry to be seen as ‘responsible’ through benchmarking or codification. They certainly mobilise resources and provide a strategic space in which industry positions can be consolidated to engage external actors, including government and civil society (ibid 50f.). Coalitions may, therefore, be pre-occupied with controlling the narrative to legitimate their collective actions, for example, by setting responsibility agendas that are impactful but limit the effect on industry business models (Du and Vieira, 2012).

Given these different possible motives, industry-led responsibility is likely to imply a limited notion of responsibility that is commensurate with shareholder value priorities and some highly codified, ‘ethics-light’ additions to the business model (Raiborn and Payne 1990). CR actions that are expected to reduce profits for any adopters will disincentivise collective action (Sternberg 2011); and other actions which may allow some firms to enhance profits may be subject to firm-level gatekeeping, not industry-level action (Doane 2005). Under some circumstances industry can organise through self-regulatory institutions, for example, as ‘green clubs’ to provide collective guidance and control behaviour of members (Marques 2017; Beschorner et al. 2017). For example, Tischer and Remer (2016) illustrate how social banking associations self-regulate conduct and work towards common goals that seek to benefit society and environment. Extension of this to the whole of the banking sector, however, is problematic because it requires agreement on what is ‘right’ and what is ‘practical’ (Raiborn and Payne 1990, p. 885). In other instances, ‘industry-led’ coalitions at the national and global scale are a response to improved coordination between civil society actors who may focus on industry directly, rather than government indirectly to enact change (Grayson and Jane 2013; Scherer and Palazzo 2011). For example, recognition by civil society that ‘target setting’ or ‘best practice’ initiatives by government encourage industry adherence to minimum standards, rather than seeking transformative action, can encourage more direct or disruptive actions to pressure companies and industries to change (de Bakker et al. 2013). Under such circumstances, it is harder for industry-led initiatives to engage constructively with more fundamental, normative notions of ethical behaviour.

### Stakeholder Coalitions as Drivers of ICR

**Fig. 1:**
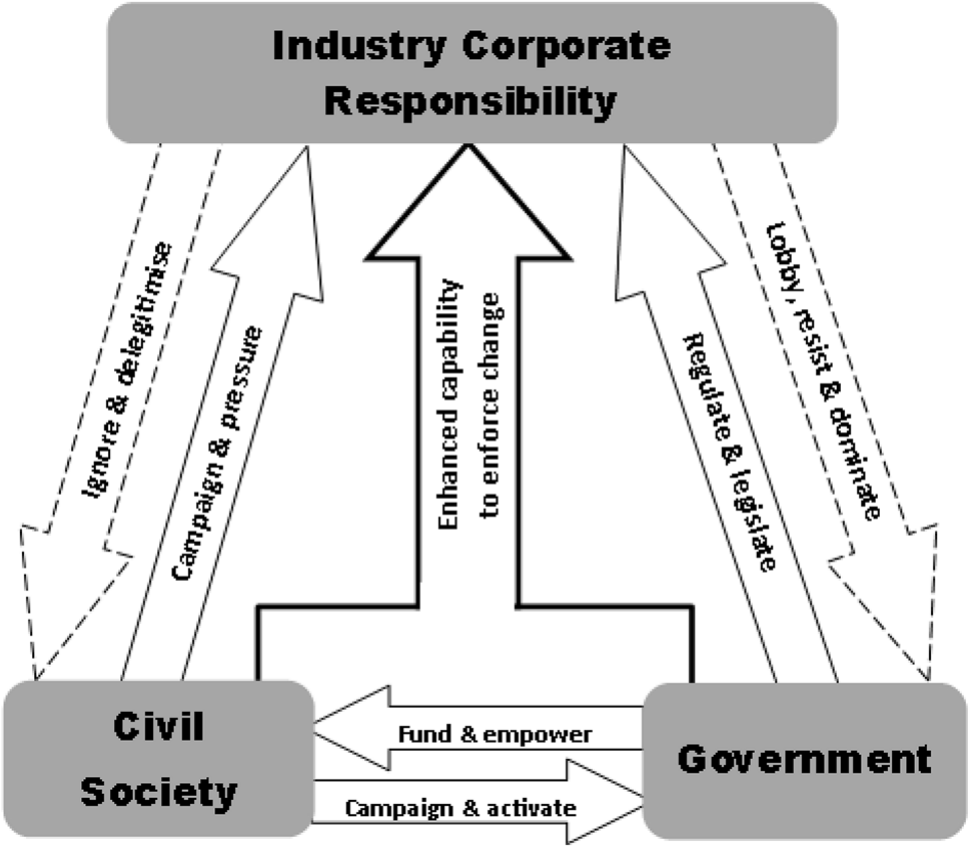
Influences on industry corporate responsibility. Source: authors

Drawing on these arguments, our framework focuses on how government and civil society can enable and influence ICR. Figure 1 illustrates key interactions or pathways between civil society, government and industry to enhance ICR, as well as sources of resistance. In response to external pressure, industry may individually or collectively engage tactics such as lobbying or reporting; while government has means to enable or to obstruct civil society’s ability to promote pressures for change (Clark 2011). Ultimately the power of either civil society or government to change industry-wide behaviour is limited (Dentchev et al. 2017; Carberry et al. 2017). For example, while civil society has developed persuasive (media campaigns, shareholder resolutions) and disruptive (boycotts, collective legal action) tactics, it lacks the ability to coerce firms directly into adopting ICR (Carberry et al. 2017). Governments at different levels often approach ICR through generic policy responses which are often fairly ineffective. The EU, for example, is integrating ‘sustainability’ into a broad financial policy framework in order to mobilise finance for sustainable growth with support from industry and investors (European Commission 2018) as well as driving industry and government alliances, to the detriment of civil society which is insufficiently included in these approaches (Moon and Vogel 2008). However, industry involvement in new sustainable finance policies could explain, for example, the slow pace of the review of the Capital Requirements Regulation (CRR2) and the Capital Requirements Directive (CRD 5), as well as the exclusion of an external evaluator in the new EU green bond standard (European Commission 2020).

Even if we share Den Hond and de Bakker’s (2007) optimism that civil society activism can influence firm behaviour directly, doing so may be more difficult when such activism targets general, rather than specific, behavioural change of an industry. This is particularly the case where change means not simply ceasing specific practices but developing a more ethical approach to future choices and activities. However, by aligning its interests with government, civil society may be capable of influencing cultural and regulatory conditions of an industry; in other words, it can create a political environment for the diffusion of its objectives (Carberry et al. 2017). The ability of civil society to re-politicise what is increasingly an attempt by government to institutionalise CR (Moon and Vogel 2008) holds government more accountable in enforcing legal obligations and societal values. In doing so, it creates a set of legitimate pressures on business to act responsibly. But, industry and government can also frustrate civil society ambitions to drive ICR: for example, industry associations can resist change while also lobbying government; or industry-government relations may become shaped by revolving-door arrangements or other forms of capture that undermine third-party initiatives, including by displacing the agenda onto more trivial or peripheral issues (Tashman and Raelin 2013). This leads to our second question: what conditions might allow some actions—including coalitions or alignments of interest—to be more effective in producing meaningful ICR?

To identify potential factors that might shape ICR we draw on Mitchell et al. (1997), Neville et al. (2011) and Ali (2017) to highlight a range of features—power, legitimacy, urgency and magnitude—which in some combination might lead to industry-level initiatives. Mitchell et al. (1997) develop a conceptual analysis of stakeholder salience based on power, legitimacy and urgency, while others propose different typologies to help explain a greater degree of stakeholder orientation in proactive firms (Neville et al. 2011; Ali 2017). In trying to understand how specific *industry-level* initiatives might come about, the framework also needs to incorporate how stakeholders (civil society and/or government) can create the conditions to promote responsibility without direct or proactive involvement of individual companies. This contrasts with an assumed need for stakeholders to capture the attention of companies or work with them directly, which is prevalent in firm-centred CR debates.

### Stakeholder Salience: Power, Legitimacy, Urgency and Magnitude

In line with the literature on stakeholder salience reviewed by Neville et al. (2011), we suggest that attempts to meaningfully influence ICR would require stakeholders to possess both power and legitimacy. Unlike Myllykangas et al. (2010), the notion of power defined as having ‘power *over’* [emphasis original] must be relaxed to include ‘power *to*’ engage others in the process given the collaborative nature of negotiation. In line with these authors’ exploration of legitimacy as socially constructed and the general perception that stakeholder involvement is ‘desirable, proper, or appropriate’, we consider that power must equally be seen in relation to the action performed and the actors involved. The contextualisation required to make sense of power and legitimacy is provided through accounting for urgency *and* magnitude. Urgency is more widely adopted in discussions of stakeholder salience (see Tashman and Raelin 2013) defined as the degree to which claims require immediate action, both in terms of time but also recognising criticality of the form within the specific context (Myllykangas et al. 2010). However, little reference is made to the magnitude of required change. Where authors focus on magnitude, they do so in terms of consequences (Brown et al. 2016) or ‘significance’ of change (Shropshire and Hillman 2007). However, we argue that magnitude of change can also usefully address the complexity of the task at hand, not just in terms of significance or consequence but also in terms of the relational work that is required as part of the negotiation. Getting a single actor to commit to sizeable change is difficult enough; but successfully enacting magnitudinal change across an industry is likely to be more difficult, especially when competitive agendas collide. Urgency and magnitude of the proposed action therefore both affect the ability to influence ICR, including attempts to develop more responsible behaviour at an industry level through processes of negotiation and coalition building.

Collaborations between civil society and government are therefore likely to be beneficial where substantive and/or immediate change is required to effect more ethical behaviour at industry level. For example, the *MoveYourMoney* campaign, initially driven by civil society actors from 2009, led to the UK government creation of the *Current Account Switching Service* (CASS) in 2013 (Seyfang and Gilbert-Squires 2019; Tischer 2013). In this case, the independent power and legitimacy of civil society and government was enhanced by collaborative efforts: when government joined the civil society campaign, the impact was scaled up from half a million bank customers switching accounts prior to the introduction of CASS to 5 million since (Tischer 2013; Pay.uk 2019). Here, successful collaboration follows individual attempts to seek influence. Initial differences are overcome as actors consolidate and extend power and legitimacy, for example, through formal electoral support from society, or less formally through campaigns. This can happen without the explicit support of the industry and even where there is active resistance reflecting concerns for the profitability of members.

Understanding CR initiatives as a process of negotiation between key actors illustrates the complexity inherent in improving responsibility at industry level and the role of different groups. Changes that result from pressure by government or civil society for change, leading to legislative or other actions, may be different to CR actions initiated by the sector. Holzer (2008, p. 59f) unravels some of the complex interactions between those seeking change (external coalitions) and the affected firms. In particular, he notes the propensity of diverse coalitions with dispersed power to assume a passive role during negotiations as control over the process is internalised by the firms’ top management. Such internalisation may also occur at the level of the industry, to either reject or accept change, facilitated by industry associations. Coalitions, however, are brittle: they may be dominated by a set of actors or they may be divided and seek to influence ICR independently, which can damage legitimacy. All of these may enable or disable action by management across the industry to resist pressure for ICR; and it may therefore limit the power by civil society and government to affect industry behaviour (Holzer 2008). In other words, management (or its representative trade body) may assume the role of political broker, enabling them to strategically manage stakeholder coalition interests or avert external influence (Campbell 2007, p. 955).

If, however, ICR initiatives are to be understood as resulting from pressure of outside parties—civil society or government—what kind of power is implied, both in making change and in resisting it? Some instances of power seem straightforward: government passes a law that requires a specific behavioural change and instructs a regulatory agency to enforce it. This results in a very narrow kind of ICR because it reflects compliance rather than voluntary recognition of inherent stakeholder interest. In the absence of voluntary action, desired changes may be sought via incentive systems or by introducing principles or frameworks that encourage better outcomes but do not compel firms to adopt or desist from any particular practices. Power here takes a more social form (Dowding 1996; Lukes 2005), which reflects the ability of an actor (such as civil society or public authorities) to entice and shape, rather than force, the actions of others (such as retail banks), thus ‘furthering their own interests and/or affecting the interests of others’ (Lukes 2005, p. 65). Power is also revealed through the extent to which one actor (here, business) can decide how (if at all) to respond to the stakeholder initiative (Lukes 1976, p. 129). In some cases, business may resist all attempts at enrollment by civil society and/or government, especially if there is no (prospect of) legal requirement or a lack of collective public interest.

The absence of industry-led CR might reflect some degree of industry power vis a vis other interests—a belief that no significant consequences may follow from inaction—as much as a lack of organisation, even where there may be legitimate concerns. Power is, however, often contingent and it may be mediated or created by specific circumstances. Business or industry perceptions about when action is necessary will also reflect the context, as civil society or government responses to evolving problems. As such, aspects of power and legitimacy afforded to civil society and government are context and time specific, not permanent or enduring attributes. Once irresponsible behaviour is corrected, actors may reorganise resources to focus on alternative issues (Roloff 2008).

The framework, therefore, needs to reflect the magnitude of the issue and the urgency with which it must be addressed; these might empower or alternatively undermine the strength of coalitions or the ability of civil society to negotiate. For example, proposed ICR actions may affect a set of firms across an industry equally, or disproportionately affect a subset of the actors by virtue of process or product characteristics. The reasons for such considerations become clear if we think about what is at stake. In pushing for new, agenda-setting ethical policies or actions that affect all players within an industry the stakes are relatively high and, therefore, may be met with more (coordinated) resistance from the industry. Seeking to influence behaviour across an industry thus may require considerable resource to be spent compared to situations in which one firm is the target of civil society and/or government action. In other cases, urgency (at least as perceived by civil society and/or government) may become a more important driver and traditional forms of negotiation involving consultation and discussion (Campbell 2007) may not deliver desired outcomes. Thus, the nature of negotiation may be issue-specific, reflecting how power to influence and resist is distributed amongst the various coalition members. ‘Power’ here is to be understood as a fluid expression of changing contexts over time, reflecting the composition of and work undertaken by the coalition. For example, initial ambitious demands can become scaled down to realise more modest change; in this way, magnitude as a driver of significant change may be undermined by an urgent need to act or a willingness to compromise with a more incremental or superficial style of reform. Or, magnitude or urgency around a particular issue can allow civil society to mobilise additional financial, political or other resources that augment power and compel change.

## Industry Corporate Responsibility Initiatives in Retail Banking: Coalitions and Public Action in Spain and the UK

Corporate responsibility reports in the banking industry have served the principal purpose of marketing to interested stakeholders, including socially responsible investors and stock market index managers. From the perspective of the industry, this might be viewed as a cost-effective way to deal with the demands of interest groups and enhance reputation, while avoiding significant and potentially costly changes to business operations and conduct. Although most banks have published CR reports as part of their annual accounts or as standalone documents before the crisis (Coupland 2006, p. 3), the quality and scope of such reporting differs considerably between banks (Novethic 2012, p. 5; Scholtens 2009, p. 4). Despite the emergence of the *Global Reporting Initiative* guidelines (GRI 2013, p. 9; Roca and Searcy 2012), there has been little standardisation of disclosures across the industry, even where specific banking reporting standards or guidelines have been issued. Crucially, qualitative reporting is unlikely to become widely established as promoters of ESG and sustainable principles tend to prefer readily quantified data—such as greenhouse gas emissions or gender equality—over qualitative data (Hiss 2013). The incorporation of ESG criteria in financial product creation further commodifies sustainability as something that is quantifiable (ibid); this is underlined in EU *Sustainable Finance Action Plan* (EU Commission 2018). Industry stakeholders are also requesting more information on what is measured and how (see Freshfields 2019), though it is unclear if this will go beyond reporting to address bank business models in relation to ESGs (Siew 2015; Lyon and Maxwell 2011).

Even without an overall framework, assessment tools (see, for example, Novethic 2012) have been used as a proxy to evaluate retail bank functions, narrowly focusing on easily obtainable parameters such as *stakeholder engagement*, *sponsorship and social engagement*, and *financial inclusion*; and some more industry-specific yet easily quantifiable discussions of *responsible lending*, *responsible marketing and customer relations.* The first three can be understood as a response to stakeholder demands and have (albeit minimal) cost implications; the latter three engage more directly with the market in sustaining or increasing customer numbers and lending, and thus are crucial to sustain profitability.

Pressure to maximise profit is deeply embedded at both cultural and structural levels and has persisted post-crisis despite public disquiet and declining returns (Froud et al. 2017). Information asymmetries between bankers and consumers, civil society and government are significant and are an indicator of industry power so that the ability to pressure banks into adopting CR initiatives is restricted and/or requires considerable resources to be spent (Ford and Philipponnat 2013; Scholte 2013). However, a greater public focus on banking following the crisis made those resources available at both government and civil society level, generating numerous publicly available reports (see, for example, on the UK: NEF 2009; CRESC 2009; LSE 2010). While much of the high profile censure has been focused on investment banking—for example, widespread market manipulation coordinated by leading international financial conglomerates caused uproar internationally—retail banking has also been characterised by industry-wide misconduct, including mis-selling products to consumers or the lack of lending to SMEs as a result of credit rationing (Dowell-Jones and Kinley 2011, pp. 202–203). Such actions have led to a lack of trust (especially of large banks) among customers (Hurley et al. 2014); and this is despite the early adoption of CR by the banking industry (Soana 2011). Not surprisingly then, the opinion poll data show that the responses to allegations of mis-selling, market manipulations and so on are directed at the retail banking sector as a whole, not simply at individual banks (Populus 2017; YouGov 2018). In this post-crisis context, ICR initiatives that have arisen can be understood as reflecting both magnitude and urgency.

To illustrate the nature and effectiveness of industry-level CR in retail banking, the paper considers two countries, Spain and the UK, where significant pressures for change by civil society and/or government can be identified to address aspects of unethical behaviour. These two countries also provide different contexts to explore post-crisis experiences in the same sector. Traditionally, Spain has a more coordinated market economy with a bank-based financial system, compared with a more liberal market economy in the UK and a market-based financial system. Jackson and Apostolakou (2010) argue that different systemic environments foster different types of coordination between business and society, thereby producing distinct models or business organisations. However, in the specific case of retail banking there are also common features in terms of how responsibility concerns have tended to be mediated through the interests of private and/or business customers. As a consequence, there could be less potential for conflict between these (customer) stakeholders and industry, compared with other industries where stakeholders may have less obvious or concentrated ‘market power’. Elsewhere, CR initiatives have covered broader concerns about the environment or human rights, where mobilising customers is only one part of the negotiations.

Nonetheless, we argue that retail banking is characterised by passivity despite the significant decline in trust noted above: public discontent with retail banking both before and after the crisis has brought little change in the nature or extent of industry-led responsibility. Instead, attempts to drive responsible behaviour come either from public authorities or are derived from civil society responses that mobilise consumer or political power. Rather than leading ethical change, industry measures can generally be a response to—or compliance with—formal or regulatory initiatives. The lack of proactive industry change may appear surprising given public discontent after high profile mis-selling and eviction scandals in the UK and Spain respectively. In the UK, the top 10 financial scandals since 2000 have cost banks £67.4bn collectively (FT 2019), yet market shares of the major players have been relatively unaffected, reflecting the perceived ‘hassle’ of switching and mimetic business models of banks (Panther and Farquhar 2004; Froud et al. 2017). To explore this further, we first explore the context in which stakeholder mobilisations took place in Spain and the UK. Secondly, in the next section, we take two examples and analyse the actions taken by civil society, the extent to which coalitions of interests have been formed and the outcomes of the initiative in terms of embedding ICR.

### The Retail Banking Context in Spain and the UK

The retail banking industries of Spain and the UK are both characterised by a lack of diversity and, despite official attempts to change this, there has been an entrenchment of large players with similar business models and a high collective market share. These are also banking systems which have been the subject of widespread concern around treatment of customers, making them interesting cases to explore the nature and extent of ICR. This section uses secondary data to outline key trends in the retail banking industries of both countries, the major issues for responsibility and the limited nature of change overall.

Spain was severely hit by the financial crisis in 2007/08. The doubling of domestic lending by the banking sector from 100% of GDP in 1995 to 215% in 2008 (Fernández-Olit and de la Cuesta-González 2014) illustrates the scale of change in banking and the economy more broadly. Much of this newer debt was linked to a property boom: the market for mortgage lending was effectively created during this time, growing from €37.9bn in 1988 to €1065bn in 2008 and equivalent to 61% of total outstanding lending to the private sector (AHE 2012). Savings banks (SBs) were the key players, with over half of the market for mortgages, and thus were most affected when the market turned in the post-crisis recession.

Some private commercial banks and, particularly, SBs required substantial government assistance in the post-crisis era; just as significant was the shrinking of the SB sector, leaving commercial banking interests the dominant force (IMF 2012b) and abolishing a regional banking market with government participation in favour of increasing stock market control. Only two small SBs survived as many were bailed-out and converted to private banks. As Table 1 shows, within a 10-year period, the Spanish banking sector moved from a diverse industry featuring more than 60 institutions with different ownership structures and a regional banking market, to an increasingly consolidated and centralised national system dominated by stock market listed banks with only a dozen institutions (EBF 2013, p. 81). The number of credit institutions fell by 43% between 2008 and 2016, and by 2018 the five largest entities accounted for over 70% of the market (in terms of total assets), an increase of more than 50% in ten years and significantly above the average for the euro zone (Cruz-Garcia et al. 2018).

**Table 1:**
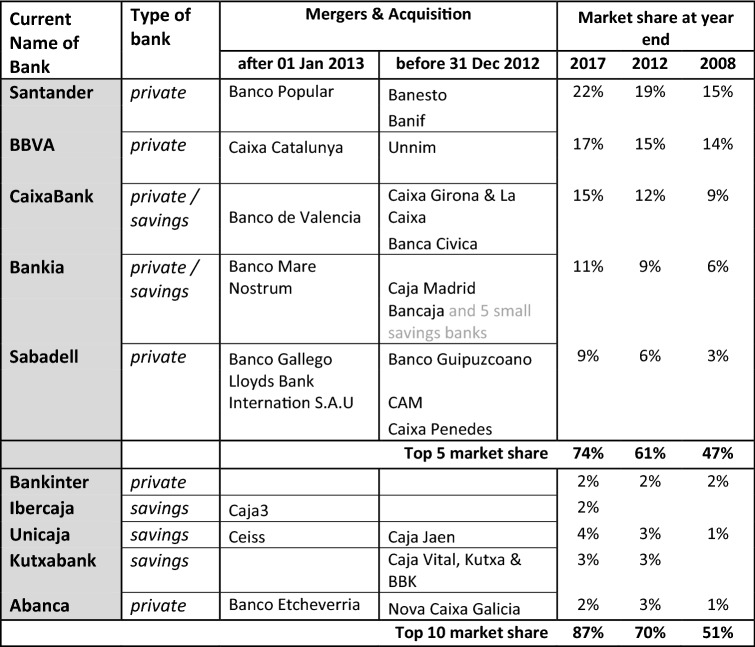
Retail banking evolution of the top 10 Spanish banks, 2008–2018. Sources: IMF (2012a, b) and Scope Ratings (2017)

Banks participating in M&A and market share in brackets

As a consequence, employees and branches have been reduced by nearly one-third between 2008 and 2015 (Maudos 2017), though Spain still has the second highest bank branch network density in the EU-28. While some reorganisation of the banking network could be deemed necessary, it was initiated in a crisis context and continued in an uncoordinated way because this covers not simply the closure of branches to reduce operating costs, as in most European countries, but also the collapse of the savings bank sector. The process of consolidation and concentration (FT 2013a) was accompanied by a move towards a more homogeneous Spanish retail banking sector, with more competition and less collaboration between the remaining larger players as banks have moved out of their traditional regional bases.

Before the crisis, government action to promote CR in the Spanish banking sector focused on legal requirements on good governance, transparency and reporting. While the post-crisis response reflects some acknowledgement of problems, it has been limited and fairly generic. For example, the Spanish government followed EU and OECD guidelines on financial education policies to improve financial literacy; and initiatives to increase SME credit through the public finance agency (ICO) have also been developed but with little effect (Ayuso 2013). More significantly, the new Savings Bank Law (26/2013), issued to reorient SBs as a locally embedded sub-sector committed to financing SMEs, had no impact given that most of the Spanish SBs had already vanished by 2013 and cannot easily be reinvented. In the absence of government requirements, banks have done little beyond ‘soft’ voluntary CR in areas like transparency and financial education. Thus, while responsibility initiatives have come from government, there has been little apparent or effective coalition building with civil society. Nevertheless, as we will see in the next section, laws have emerged to protect customers from aggressive practices in retail banking as a response to civil society pressure and judicial judgements.

**Fig. 2:**
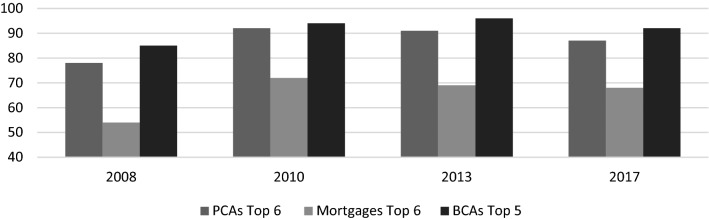
Concentration in UK retail and SME Banking (market share in %), 2008–2017. Sources: CMA 2016; FCA 2018. Notes: SME account concentration for England and Wales only [Scotland and Northern Ireland BCA markets are historically even more concentrated (https://publications.parliament.uk/pa/cm201415/cmselect/cmtreasy/204/20407.htm)]; BCA data for 2017 are estimates

In contrast, the UK retail banking sector was characterised by a high degree of concentration in a few global financial conglomerates well before the crisis (Cruickshank 2000). Despite calls from government and elsewhere to increase competition, concentration has persisted in major retail banking markets (see Fig. 2), especially in personal current accounts (PCAs), mortgages and personal loans (ICB 2011, p. 166). In fact, crisis-related consolidation increased concentration in the UK market: the six biggest retail banks had a market share of more than 90% in 2010, and have lost less than 5% since (FCA 2018). SME accounts and lending have historically been even more concentrated: the top five retained 83% of SME current accounts (CMA 2016), despite a recent increase in the number of challenger banks, including fintech. If these new banks do gain significant market share from the big players, it is not clear that this will encourage more responsible behaviour. Arguably, the attention on cost reduction is likely to continue (including further branch closures) while the large banks seek to imitate the digital technologies and product innovation and seek acquisitions of successful new entrants (Knight Frank 2018).

As in Spain, reduced diversity is also a feature of the UK retail banking industry. This started in the 1970s with the opening up of the residential mortgage market (to break-up the ‘monopoly’ of building societies), continuing with the demutualisation of the largest societies and their subsequent take-over by established publicly listed banks before and after the crisis. In addition to this external threat, building societies have consolidated their activities through a series of mergers, reducing the number of active societies from 481 in 1970 to 44 in 2017 (BSA 2018) and creating one large building society, Nationwide (holding more than 60% of sub-sector assets). Credit unions which flourished in the US, Canada and Ireland, have never moved beyond niche status in the UK, despite some modest growth (Bank of England 2019).

In the UK, civil society has exercised voice by lobbying for and responding to public inquests using two channels. First, civil society organised immediate (G20 protest in 2009) and more sustained (Occupy London) forms of protest, as well as public campaigns for change (MoveYourMoney; The Robin Hood Tax, Positive Money) and alternative economy think tanks such as NEF, RePublica and Demos (see, for example, Positive Money 2012). But dissatisfaction with post-crisis reform was soon absorbed into the wider anti-austerity movement as the long-term socio-economic consequence of the GFC outgrew the ‘financial crisis frame’ (Pianta 2013), leading to more direct engagement with the policy process. Second, although often excluded from policy making at a formal level, civil society groups have submitted responses to regulatory initiatives, for example, in response to the *Independent Commission on Banking* (Positive Money 2012; Ford and Philipponnat 2013). In Europe, civil society has established *Finance Watch* in 2011 to ‘act as a public interest counter-weight to the financial lobby’ (ibid: 188).

In terms of post-crisis responsibility in the UK, public bodies (regulatory agencies and government) have been the main source of initiatives, reflecting frustration about the limited changes to culture and behaviour from early and sustained civil society action. The rediscovery of regulation is in marked contrast to the previous explicit commitment to the deregulation of financial services since the mid-1980s (Moran 1991) and, albeit short-lived, encouragement of business-led CR using a market-based approach (Shaefer 2013, p. 243). While there have been industry-led programmes in financial services (such as around financial literacy), there is little evidence that these have led to significant changes in behaviour and business models (Froud et al. 2017). Banking crisis, and the ensuing unprecedented government intervention to support the sector, has led to a marked rebalancing with changes to the regulatory framework and a more active interest in retail banking by government, especially to increase competition.

Government-led ICR initiatives have taken three forms: promotion of new entrant—challenger—banks; encouragement of lending to SMEs; and the principle of ‘treating customers fairly’ (considered in the next section of the paper). The first of these rests on the assumption that promoting competition is the mechanism to encourage existing retail banks to treat their customers well; this is a priority that has led to a succession of investigations into the structure of the UK retail banking sector, the most recent of which reported in 2016 (CMA 2016). The encouragement of challenger banks—including offering substantial financial support to some new entrants under a *Capability and Innovation Fund*^2^ and simplifying the process of obtaining a banking licence—represents an attempt to reshape the market. Unlike in Spain, however, there is no explicit preference for different kinds of banks such as savings banks. While a number of new banks have entered the PCA and business markets, this has had limited effect on the overall banking structure as many of these are small and present niche offers; indeed, a process of consolidation of new banks is already underway (English 2019).

The second main government objective, to foster lending to SMEs, has led to initiatives directed at existing banks, such as ‘Project Merlin’ launched in 2011. After this project failed to meet targets (Independent 2012), a new initiative ‘Funding for Lending’ ran from 2012 to 2018 (Bank of England 2012)^3^ with similar results. Despite this, the limited extent of lending by banks to SMEs remains an official concern, and a new venture, the Business Bank, was established in 2013 with direct government support (FT 2014). Overall, UK government actions to encourage more responsibility across the industry have taken various forms including financial inducements and projects but these have largely not involved effective coalition building. Within these broader national contexts, the next section considers two examples which show a greater degree of coalition building reflecting combinations of power, legitimacy, magnitude and urgency and mobilising new and existing organisations.

## Industry Corporate Responsibility Initiatives: Two Examples of Coalitions and Public Action

Following our theoretical framework, we present two emblematic examples to analyse how the magnitude and urgency of industry bad practices provoked coalition building initiatives from civil society and changes at industry level. These were selected because they reflect important and legitimate issues where banks have acted against the interests of their own customers. In both cases, civil society action was central in helping to collectively organise the interests of individual customers and in doing so increase the urgency of the problem and the legitimacy of the action; moreover, civil society action prompts some form of response by government and other public authorities which is necessary to effect change. In each case, we describe the problem, the characteristics of the negotiation process, the actors involved, the reaction of public bodies and industry and the limitations of this process, taking into account the different contexts.

The first case, protecting mortgage debtors in Spain, is a significant and unprecedented example of mobilisation of power given Spain’s low level of consumer activism. In this instance, a coalition was built between bank customers with mortgage arrears and other citizens concerned with social justice. The bottom-up coalition drew power and legitimacy from the magnitude of the problem (around half a million households facing or experiencing eviction by their banks) and the urgency that relates to the potential and visible harm that eviction can cause, especially to vulnerable people. PAH, as a new, civil society organisation worked effectively and persistently to exert pressure on banks via judicial action and, later, government action. The direct outcome is changes in bank behaviour in relation to these affected households, but this has not extended to any more general embedding of responsibility. There may, however, be more enduring effects through the demonstration of effective civic action, making future stakeholder coalitions more likely.

The second case, the response to mis-selling of financial products by banks to UK customers, was made urgent through the action of an established, well-resourced civil society organisation, Citizens Advice. This organisation used legal process to challenge government inaction in response to massive mis-selling which affected millions of bank customers. While the failure of an insurance policy on a financial product such as a loan is perhaps less serious for the individual customer than a potential eviction, the sheer scale in the second case provided legitimacy. The successful legal action rebalanced power away from the banks in relation to this issue and required government to align itself more explicitly with customer interests. Again, this has been a high profile and extended action, yet the extent of responsibility has been limited to specific redress, not behavioural change by retail banks. Nor has it shifted government policy in ways that prevent future mis-selling.

These cases are developed using secondary data to inform understanding of the nature and scale of the process, the actions taken by civil society, formal responses by government and public authorities and the perspectives of citizens, as set out in the media of different kinds. In each case, the secondary data are used to trace the development of the problem, the critical interventions by civil society and the forms of response by government and other public authorities, including aspects of coalition building, and the extent of short- and long-term change. The major sources for the Spanish case are statistical data from CGPJ (The Spanish National Council on Judiciary), official court reports, PAH reports and media articles. UK data are drawn from regulatory authorities, Citizens Advice, media and other commentaries.

### Example 1: Protecting Mortgage Debtors Against Repossession in Spain

This first example is concerned with the relationship between banks and residential mortgage customers: the effectiveness of coalition building is a consequence of broadening the issue beyond a simple market relation to invoke the wider social justice implications of bank behaviour, which was critical to establishing legitimacy. What is also striking about this example is that civil society was able to co-opt judicial processes to more effectively bring government into alignment. However, while there are demonstrable changes in bank behaviour in some respects, as outlined below, there is no clear evidence of underlying ethical shifts in banking practice led by the industry.

Lending practices in Spain before the crisis increased home ownership. After the crisis, however, rising unemployment contributed to the growing number of mortgage holders unable to meet payments, resulting in mass evictions by the banks: an estimated 500,000 releases or evictions occurred between 2008 and 2013 (PAH 2014). Public outcry about the social consequences of eviction and homelessness led to the establishment of a civil society movement, the Spanish Platform of People Affected by Mortgages (PAH) in 2009, which campaigned for more considerate treatment of distressed debtors. PAH is notable as a social movement because it changed the framework surrounding the eviction problem, while also creating nationwide awareness of the resulting injustice. De Weerdt and García’s (2016) analysis of this innovative social movement found that PAH empowers people through solidaristic actions to place collective pressure on legislative bodies, governments and banks. Newly established collective communication channels enabled these groups to reach agreements with banks to renegotiate or even condone debts, or to extract socially beneficial terms from banks, such as payment in kind or a social rent. These authors conclude that PAH has been successful in arguing that the debt problems experienced by Spanish households are not the result of individual failure but a consequence of business decision making and practices that put profit ahead of the consumers.

The success of the campaign against evictions reflects coalition building around an urgent issue. PAH enlisted the EU Court of Justice, which ruled against the evictions and gave Spanish courts new power to stay evictions (FT 2013b). Resulting legal and social pressure led to the issuance of the Royal Decree Law 6/2012—a mechanism that allows urgent laws to be passed—to protect mortgage borrowers. This introduced a series of measures, including a voluntary Code of Good Banking Practice which included exceptional measures for the resolution of defaults of people in difficult social conditions. A subsequent law (1/2013) and amendments formalised detailed measures to protect mortgage debtors, debt restructuring and social rent, and thereby recognised the social consequences of eviction which had been initially ignored by national government.^4^ In an attempt to prevent future bad practice the law also promotes more responsible lending, limiting mortgages to a period of 30 years and a maximum loan-to-value of 80%. However, the law does not universally and retrospectively alter payments as PAH had argued for, though it includes a two-year suspension of evictions in cases of ‘special vulnerability’. As a result, property can only be repossessed and sold when restructuring is impracticable under specific conditions, according to the Code of Good Banking Practice.

The PAH example illustrates social innovation by organised citizens fighting for social justice. The coalition has incorporated support from 200 collectives and labour unions, more than 100 city councils which have approved motions in favour of the demands of PAH and from political groups in 14 regional parliaments across Spain (Bhandar 2015). This has produced political action which has shaped governance through creation of new mechanisms for collectively negotiating housing debts with financial institutions, while also influencing new local and regional regulations for access to housing for mortgage victims. By reframing the issue as a collective problem—governments have used an ‘individual approach’, the PAH has focused on collective responses to social problems—PAH’s campaign contributed to new ways of conceptualising and approaching policy problems (Pradel et al. 2013). The success of PAH is underlined by subsequent legal action brought against Blackstone, the largest owner of housing in Spain with some 40,000 homes. PAH has accused Blackstone of committing a crime of ‘hoarding’ empty homes and ‘deceptive alteration of the price of things’—buying repossessed homes at a low price and then withdrawing them from the market to sustain high rental values and purchase prices—which are illegal (PAH 2018a). This is a particularly sensitive political issue as social housing for rent provides only 1% of Spanish homes (compared with around 20% in the United Kingdom, France or Germany) and there are 3.4 million empty homes.

While a remarkable and unusual example of organised citizen action, it is important to ask whether this has enhanced responsibility more generally in Spanish retail banking. The effectiveness of PAH’s social action and heightened awareness of bad practice has encouraged consumer associations and lawyers to engage in a context where traditionally there has been limited consumer mobilisation. However, it is not clear that corporate behaviour has changed more generally. For example, between 2007 and 2018 there were more than 500,000 evictions in Spain due to mortgage non-payment and, in recent years, this figure has been increased by rent evictions (PAH 2018b). Rather than preventing future unethical behaviour, further bad banking practices have emerged. The insertion of floor clauses in mortgage agreements, for example, meant that consumers would not benefit from reductions in EURIBOR rates, but were liable to pay more if this rate went up. This was deemed an ‘abusive practice’ by the courts and a 2017 royal decree established an extrajudicial channel to settle disputes (BOE 2017) and by end of 2019, over €2.2 billion was returned to Spanish consumers.^5^ These and other practices have undermined public trust in banks, with significant judicial proceedings still awaiting resolution (Spanish Central Bank 2019). Overall, this case demonstrates that banks’ reaction to civil society pressure may not result in any generalised corporate responsibility but can have issue-specific consequences. Business model pressures remain focused on a single-bottom line and civil society initiatives lack the (legislative) muscle to drive meaningful and far-reaching changes in corporate behaviour across an industry. Government action was necessary to achieve the outcomes supported by civil society and, later, the judiciary, though there was no immediate response by government to enact structural policy changes to prevent poor behaviour by banks.

### Example 2: ‘Treating Customers Fairly’ and Mis-selling in the UK

Treating customers fairly (TCF) has been an officially endorsed general principle in the UK since 2006 (Shaefer 2013), applying not only in banking but in other utility sectors like energy. TCF in retail banking was renewed post-crisis after a review of the regulatory framework on financial services and the creation of the new regulator, the Financial Conduct Authority (FCA). TCF encapsulates a basic ethical principle to which all organisations regulated by the FCA must adhere: a bank ‘must pay due regard to the interests of its customers and treat them fairly’. If the principle is embedded in bank behaviour then it should deliver six outcomes which collectively represent ‘fair’ treatment (FCA 2013). This is not a regulatory framework, nor a set of benchmarks for behaviour; enforcement is via an Ombudsman and investigations when things go wrong. However, under a self-interest argument, it might be expected that banks would have an interest in following TCF to ensure that (mis)treatment of customers does not contribute to future scandals, reputational damage and fines, as well as contributing to rebuilding trust in the retail financial services sector.

The survival of TCF as a regulatory initiative reflects the continued need for improved transparency and fair treatment, rather than its success in enhancing and enshrining responsibility. The renewal of TCF in 2013 was amidst a series of mis-selling scandals, most notably of payment protection insurance (PPI), an insurance policy sold by banks and other financial institutions linked specifically to debt and credit products. The scale of mis-selling suggests a mass failure by the sector to follow the TCF ethical principle (and a failure of public authorities to take remedial action). Moreover, this was not the first instance of mis-selling by UK banks. Earlier mass episodes of unacceptable behaviour in relation to selling private pensions and endowment mortgages in the 1980s and 1990s (Ferran 2012) had not reformed the incentives systems and culture within banks which allowed serial mis-selling to thrive. The original failures of mis-selling were compounded by the way that banks dealt with customers who complained. As a result, fines were imposed by the FCA on some banks for mis-handling complaints (see, for example, FCA 2015).

In the specific case of the mis-selling of PPI, the interests of customers were taken forward by Citizens Advice—a network of charities that provide free legal information and advice—which became increasingly aware of the large number of policy holders who had made claims under their policies and been refused pay-outs (Citizens Advice 2015). As PPI products became more widely sold, it was more likely that they were unsuitable or at least represented very poor value. Citizens Advice noted in 2005 that there were estimated to be 20 million live PPI policies with an annual premium of £5.3bn, compared with £8bn and £9.5bn, respectively, for property and motor insurance. Through their individual charities they observed the massive extent of customer dissatisfaction, though this was disputed by the industry. In particular, it appeared that lower income and vulnerable customers were more likely to have been mis-sold these insurance products (Citizens Advice 2005).

Citizens Advice collected individual cases to make a ‘super complaint’ to the regulator, the Financial Services Authority, in 2005. Public authorities responded to this civil society action by putting in place a system of fines and compensation for customers who could demonstrate they had been mis-sold PPI. Far from recognising their responsibilities, banks via their trade association the British Bankers’ Association (BBA) initially mounted a legal challenge to the requirement to pay compensation and indeed continued selling these products for years. In 2011, the BBA lost a legal case against new rules intended to prevent mis-selling, which were to be applied retrospectively, hence opening up further claims from customers (Guardian 2011). In the end, the industry had no option but to pay the regulatory fines and customer compensation. UK retail banks created enormous provisions in their financial accounts to meet the compensation costs^6^: by 2019, some £36bn had been paid out by British banks (FT 2019). The issue of corporate responsibility is relevant in two ways: the first is the sale of the financial product by banks, including whether it was suitable for the customer and clearly explained to them; the second is how banks handled the complaints of mis-selling. To some extent, the second issue gained a heightened significance in some of the reporting by public authorities, perhaps because this could be measured and audited more easily than the more fundamental concerns about how banks treat customers. For example, the FCA notes in 2014 that firms have improved the way that they handle complaints, though does not comment on whether selling practices are more ethical (FCA 2014).

The regulator has since banned certain products and attempted to address selling practices more generally. However, a good test of industry-led responsibility might be whether bank business models have become less reliant on cross-selling (often unsuitable) financial products, thereby embedding the principle of fair treatment of customers into routine practices. While banks have withdrawn some products, as required by the regulator, evidence of further mis-selling has followed the PPI scandal, suggesting that TCF is still not being adopted by banks. For example, in late 2013, Lloyds Bank was fined £28 m and expected to pay out £100 m in compensation after a new expose about mis-selling a range of financial products by staff (Guardian 2013). It is no surprise that claims management has become an industry in its own right: these companies are looking out for new potential sources of mass claims, including mortgages (again) and packaged current accounts which offer a bundle of services in return for a fee (FT 2019; Which? 2018; Francis 2019).

In this example, civil society action was central to addressing the massive consequences of mis-selling, by helping to collectively organise the interests of individual customers and in doing so increase both the urgency of the problem and the legitimacy of the action. More broadly, the sustained failure of UK banks to improve their treatment of customers has provided an interesting opportunity for social movements to raise public awareness. For example, *Move Your Money* claims some success in encouraging switching away from the big banks; though the impact on their business models or behaviour is uncertain. In essence, the initiative represented a market-based approach to irresponsibility, with consumers encouraged to exercise their power by changing their bank.

To sum up, this section has analysed two initiatives where actions led by civil society, with responses from government and other public bodies, have had some impact on the banking sector. In both cases, action to address a lack of corporate responsibility is the response to the results of coalitions formed around banks, not a spontaneous act or corporate priority. To the extent that ICR has been enhanced, it is reactive not embedded in bank practices. In the Spanish example of mortgage holders facing eviction, civil society pressure has worked through public authorities to convert social concern into regulatory requirements which strengthened protection for borrowers. In the UK example of responses to widespread mis-selling, civil society action had the effect of raising the profile and achieving redress for individuals via massive compensation, but without necessarily changing bank behaviour. The implications of these examples are explored in the final section.

## Discussion and Conclusion

This concluding section reflects on the initiatives discussed in the paper and their implications for understanding corporate responsibility at an industry level. Here we come back to our theoretical framework to highlight several issues arising from the empirical analysis that can be used to frame subsequent research and to inform policy. The paper set out to address the question: whether and how public action (via civil society and/or government) can meaningfully affect industry-wide corporate responsibility behaviour by retail banks. Focusing on Spain and the UK, we have provided an overview of developments in this sector since the GFC to highlight the very limited extent of industry-led corporate responsibility. Two examples which represent exceptional cases of civil society action making problems visible—and to some extent mobilising government and other public authorities—have been analysed to explore the ways in which such action reflects power and legitimacy of actors and the urgency and magnitude of the issues in the building of coalitions around banks. The examples were chosen to highlight civil society responses to responsibility problems across an industry, as well as the importance of some follow up through policy or regulatory change. Through these we demonstrate the value of focusing on corporate behaviour at the industry rather than the firm level.

The banking industry examples analysed have allowed us to explore the cultural business ethics perspective (Beschorner and Hajduk 2017) in which the industry is a frame for actors linked to each other by a web of shared beliefs and network-like relations, and with apparently similar understandings of CSR (the materiality of issues, the legitimacy of stakeholder demands, and the role of governments). Within an industry context, responsibility can be substantiated and thus made clear and manageable for companies and their stakeholders. Retail banking is an appropriate case to illustrate the significance of responsibility initiatives at industry level, particularly in relation to the inadequacy of firm-level CR to prevent or respond to problems. The financial crisis and ensuing challenges to business models added urgency and magnitude in ways that could potentially unsettle established behaviours and strengthen legitimacy of civil society organisations. As argued in the paper, retail banks have a longstanding engagement with company-level CR but this has not prepared the industry for dealing with the consequences of irresponsibility or unethical decisions through initiatives or preventative actions.

The analysis shows that in these retail banking cases, ICR came about through coalition building by stakeholders. It is evident that proactive or industry-led corporate responsibility was limited to more trivial issues (Tashman and Raelin 2013) and there was little evidence of any incentive for industry collective action (Sternberg 2011) to initiate change. Instead, banks have been compelled to act following the mobilisation of power through legal processes and (eventually) the enrolling of governments. Overall, the analysis in this paper has illustrated how corporate responsibility at the sector level in retail banking is, first, the product of context-specific processes of negotiation between the sector, civil society and public authorities, on behalf of customers and other stakeholders; and, second, has only limited momentum in enabling behavioural change beyond the initial catalysing events.

In both cases, and particularly so in the Spanish case, coalitions are created in highly politically charged contexts. Set up in response to widespread bad practice and government inactivity, PAH was initially unable to claim legitimacy. Nonetheless, the scale of the problem addressed and the public interest created soon established PAH as a legitimate civil society actor, able to legally challenge both governments and banks. PAH effectively created a political environment which allowed the diffusion of its objectives, in line with the argument of Carberry et al. (2017). This pivoted around the idea that evicting mortgage debtors was morally and politically unacceptable. In the UK case, the issues were clearly less politically charged, yet the sheer scale of the issue—the millions of bank customers sold inappropriate insurance products—created a legitimate political force based on evidence of unethical practices. Here, our analysis supports Myllykangas et al. (2010), who argue that legitimacy has to be socially constructed, rather than arising from priorities set by government or industry. The post-2008 campaigns against banks are to this extent reflective of broader movements about capitalism, inequality and climate justice, as well as the specific unethical practices.

Our examples support the argument that attempts to meaningfully influence ICR require stakeholders to possess both power and legitimacy (Neville et al. 2011). Civil society has limited direct power but can access it through actions and coalitions: in both examples, civil society stakeholders derived power through collective action. The UK example showed how mis-sold customers were brought together by Citizens Advice, a civil society organisation with legitimacy based on longstanding efforts on behalf of consumers. In the Spanish case, action came about through creation of a pressure group with wider membership to campaign initially for mortgage debtors; subsequently, PAH has been the basis of wider campaigning on issues relating to rights to housing.

Such collectivisation is critical because, although retail banking customers are legitimated stakeholders who should be able to exert pressure on business to act responsibly, it is difficult to articulate individual power for many reasons. First, information and resource asymmetries between banking institutions and customers reduce individual’s power despite the ethical legitimacy and magnitude of their demands. Customer financial illiteracy and mimetic practices of most retail banks also constrain the ability and opportunities of individual customers to negotiate with banks. Even when they are aware of their rights, the costs of pursuing them will be an obstacle to individual remediation. However, our examples show how under specific conditions, the magnitude or scale of unethical practices provides opportunities to collectively organise, create visibility for the issue and accumulate pressures for subsequent—albeit limited—action by government and other public authorities. The ability of civil society to formalise claims of misbehaviour then creates opportunities to effectively engage in negotiations around remediation, despite resource limitations. As we have seen, the magnitude and urgency of customers claims and demands (e.g. see Mitchell et al. 1997) have allowed civil society to mobilise additional financial, political, legal or other resources that supplement individual power and compel change through the intermediation of regulatory or other changes. These were the main conditions that allowed effective actions through a process of negotiation and coalition building in our examples. Thus, civil society action was central to addressing the massive consequences of mis-selling, by helping to collectively organise the interests of individual customers, represent them in legal processes and in doing so increase the urgency of the problem. It should be noted, however, that even though initiatives led by civil society can lead to changes in the way industries are required to act, this does not imply that government priorities or objectives are necessarily aligned with those of civil society stakeholders. Where they do exist then, stakeholder coalitions are temporary and contingent.

In understanding how stakeholder pressure works, we can draw a distinction between consumer and social pressures. Consumer pressures involve those directly affected by irresponsibility such as mis-selling, whereas social pressures reflect a broader constituency: for example, PAH is not simply comprised of those who are or might be evicted but represents a more general discontent about bank behaviour *on behalf of* affected citizens. This would seem to be a stronger and extendable basis for a coalition of interests as it presents problems as a general failure to meet citizen needs, rather than harms done to individual customers. Coalition building here responds to ethical issues which relate to housing in this specific instance but could equally cover access to energy, water and sanitation, transport, legal aid or other essential material and social infrastructures. When the unethical behaviour of banks or other industries impacts on citizens’ welfare or even their human rights, corporate responsibility becomes a political issue and legislative, executive and judicial powers can be used to guarantee or at least support these rights. In these instances, it is social pressure through established or new civil society organisations rather than individual pressures, which are effective. Here, we can draw another interesting comparison. While PAH in Spain initially lacked legitimacy as a new organisation seeking power to change bank behaviour, Citizens Advice in the UK had more immediate credibility, as well as legal and other resources to draw on. Yet, Citizens Advice is also limited in scope by its success in advocating for consumer rights, while PAH can develop innovative responses to institutional failures through creating new stakeholder coalitions outside its original ambit. While the specific nature of the civil society demand and the context in which problems arise are likely to be important, the negotiating capacity of the actors is a driving force. As a result, PAH was able to make evictions an issue that could mobilise legal and political response even while other contemporaneous civil society demands, such as around public health and education, climate change or tax reforms, did not deliver the same level of success.

Notwithstanding these distinctions, in each case responses by public bodies have been effectively mobilised through the accretion of pressure, with regulatory or policy action triggered by an event (e.g. the super complaint on PPI mis-selling). Effectiveness here is dependent on the ability of civil society to draw on legal or regulatory processes that governments cannot ignore. But, even where reactions to industry-level problems have been effective, they have taken time to initiate and some have attracted resistance. In this sense their development reflects processes of negotiation that take place outside the sector, and indeed in the PPI compensation case, legally challenged by retail banking interests. In the Spanish case, the judicialisation of the process and the role played by the courts have transformed individual claims into societal and political claims with regulatory reforms. As for the banks, the industry response can be characterised as reactive. In both cases, banks fought both civil society and regulators to limit their financial responsibilities until the point where they had to comply and, in the UK example, face unprecedented costs. Interestingly, these defeats were followed neither by explicit actions to regain customer trust nor attempts to strategically manage stakeholder coalitions to limit external influence (Campbell 2007, p. 955). In this sense, the sustained failure of UK and Spanish banks to improve their treatment of customers (borrowers and savers) has provided an interesting opportunity for social movements to raise public awareness.

While both examples discussed in the previous section have had a high political and media profile—and have led to extensive redress—there is no indication that they will solve the underlying problems of unethical behaviour. The limited effectiveness of the initiatives can be partly explained by the reactive processes that deliver them and by the scale of the challenges. To some extent this reflects the underlying effective power of the banking industry in relation to both government and civil society; but it is also a consequence of broader socio-economic issues. What then are the policy implications of this analysis?

The calls for changes to retail banking in Spain and the UK discussed in this paper reflect significant socio-economic issues central to the functioning of a banking system and requiring coherent action across the sector, rather than company-level discretion. Our analysis shows that banking does not necessarily or effectively fulfil its intermediary function to support citizens and the (productive and social) economy (Fernández-Olit and de la Cuesta-González 2014). For example, banks over-lent to households in Spain to buy houses, while they have under-lent to SMEs in the UK. Retail banks have also failed to design and market products that meet the needs of customers in transparent ways, especially given many customers’ lack of financial literacy, asymmetrical information and inertia.

It is possible, of course, that episodes such as those we have analysed in this paper could eventually contribute to the development of industry-led corporate responsibility that recognised stakeholder interests and avoided such unethical practices in the future. Such action would obviate the need for regulatory responses by public authorities and would avoid extended, high-profile legal processes aimed at seeking redress for specific customers, resulting in substantial financial penalties. From a self-interest argument, our examples might suggest that retail banks *could* change behaviour to avoid repeated reputational and financial damage. To this extent, policy implications would be limited perhaps to the importance of safeguarding and promoting opportunities for civic action to support accountability. This would imply that the industry assumes stakeholder interest as a goal itself and not as a means to other ends.

Our analysis would suggest caution, however, in assuming such an outcome because of the persistence of shareholder value as a corporate objective for most banks (Froud et al. 2017). The Spanish and UK examples provide different contexts for the enactment of shareholder value: in Spain, rapid changes as the sector has moved towards a less diverse retail banking ecosystem provide an unstable backdrop to high-profile stakeholder initiatives; while in the UK, mis-selling reflects long-running problems in a retail banking sector where shareholder value-driven business models have dominated for 20 years. Though the historical context is different, changing bank behaviour in both countries is made difficult by the imperative for financial returns in business environments that are more challenging. This relates not simply to normative notions of ethical behaviour but to questions of practicality and attainability (Raiborn and Payne 1990, p. 885). There has been recent debate more broadly about the importance of long-term corporate objectives and meeting the needs of stakeholders more broadly, as illustrated by the US Business Roundtable restatement of the purpose of the corporation (Business RoundTable 2019), but there is little evidence as yet that this presages significant change in business models.

In such an unpromising environment for progressive policy, several possibilities can be highlighted. First, different behaviours may be more likely to come through encouraging new kinds of banks (e.g. mutuals) which offer customers a different model; or through controls on banking which more vigorously protect customers from unethical behaviour, rather than relying on compensation after the event. In the case of retail banking, development of a more relational business model in which the needs of customers can be met with appropriate products and services and delivered with better advice and accurate information may help avoid actions that disadvantage customers (Fernández-Olit and de la Cuesta-González 2014). Digitalisation processes in the banking industry could help in principle as banks have access to large amounts of data, which allows them to offer personalised products and services to customers to meet their financial needs and not just business objectives (García Montalvo 2014). But digitalisation processes will not necessarily improve transparency and the quality of financial advice and marketing. Some early scandals involving fintech firms suggest that these new actors have picked up ‘some bad old habits’ displayed by larger incumbents (FT 2016).

Second, government cannot abdicate corporate responsibility to (product or capital) markets, but equally, coalitions with civil society are important in directing government and holding it to account. The examples in this paper illustrate a form of negotiated power with government responding to civil society concerns. Government also have their own objectives which may direct bank behaviour in particular ways, such as supporting lending to SMEs to encourage growth, or promoting residential mortgages to expand home ownership, just as they may have reasons why they avoid tougher legislative and regulatory frameworks.

These kinds of mediation and capture are likely when the underlying issues like evictions are fundamentally political and government must be seen to respond to public outrage. The challenge for civil society is to align its interests with government, to create a political environment for the diffusion of its objectives (Carberry et al. 2017) and to hold government to account in terms of both legal obligations and societal values. Moreover, CR initiatives arising from stakeholder coalitions can only be a first step towards a more ethical banking sector where the underlying causes of the problem are complex. For example, an absence of social housing for rent or a well-regulated private housing sector may encourage high-risk mortgage lending by banks. In this context, there is a limit to what any kind of banking responsibility initiative can deliver if there are endemic problems social such as inadequate housing which requires separate action by government and other public authorities. This underlines the significance of our analysis beyond banking to other sectors where corporate behaviour both directly affects the quality of everyday life for many citizens and may be part of wider institutional limits. In this sense, promotion of ethical behaviour by business may require broad stakeholder coalitions to highlight underlying problems and social priorities. The relevance of these problems and priorities is stronger than ever in the post-Covid-19 economic and financial context. Moreover, there should be opportunities to explore the capacity of actors to influence and change industry behaviour both individually and through coalitions.

## Abbreviations

BBA: British Bankers Association (now UK Finance)
CR: Corporate responsibility
CSR: Corporate social responsibility
FCA: Financial conduct authority
GFC: Great financial crisis
ICR: Industry corporate responsibility
MYM: Move your money
PAH: Plataforma de Afectados por la Hipoteca (Platform of People Affected by Mortgages)
PCA: Personal current account
PLC: Public Limited Company
PPI: Payment protection insurance
SB: Savings bank
SME: Small and medium size enterprises
TCF: Treating customers fairly

## Footnotes

1 Industry political donations in the US illustrate actions that seeks to protect ability to generate profits over responsible behaviour. This is particularly prevalent in finance, energy and transport who fund political campaigns (OpenSecrets, 2019) seeking to avert regulation despite strong evidence that doing so harms society at large.

2 This fund has £425 million held in trust at the Bank of England and administered by Banking Competition Remedies Ltd to be spent in four rounds or pools to support challenger banks that explicitly target SMEs. This fund was established after agreement between the UK Government and the European Commission to implement the £775 million RBS state aid alternative remedies package, resulting from the bailing out of RBS and the subsequent inability to sell Williams and Glyn Bank. See https://bcr-ltd.com/wp-content/uploads/2018/09/Banking-Competition-Remedies-media-factsheet_final.pdf for more details.

3 This was supplemented by the Term Lending Scheme from 2016–2018 which underwrote an additional £15bn of lending, triggered by the Government’s attempt to boost the UK economy in the pre-Brexit period. https://www.ft.com/content/dcd16a09-1726-3904-909b-a0195f205477.

4 For details, see Law 1/2013 Measures To Strengthen The Protection Of Mortgage Borrowers, Restructuring Debt And Social Rental, Royal Decree Law 1/2015 of 27 February and Royal Decree Law 5/2017 of 17 March.

5 See: https://elpais.com/economia/2019/12/11/actualidad/1576098707_974322.html.

6 Between 2011 and November 2013, the banks paid out £13.4 bn in compensation with total provisions reaching nearly £20 bn in early 2014 (FT 2014). The total cost of compensation for PPI is estimated at £34.2bn by March 2019 (FCA 2019).
